# Protein kinase C regulates organic anion transporter 1 through phosphorylating ubiquitin ligase Nedd4–2

**DOI:** 10.1186/s12860-021-00393-3

**Published:** 2021-10-18

**Authors:** Zhou Yu, Chenchang Liu, Jinghui Zhang, Zhengxuan Liang, Guofeng You

**Affiliations:** Department of Pharmaceutics, Rutgers, the State University of New Jersey, 160 Frelinghuysen Road, Piscataway, NJ 08854 USA

**Keywords:** Organic anion transporter 1, Drug transporter, Regulation, Protein kinase C, Phosphorylation, Nedd4–2, Ubiquitination

## Abstract

**Background:**

Organic anion transporter 1 (OAT1) is a drug transporter expressed on the basolateral membrane of the proximal tubule cells in kidneys. It plays an essential role in the disposition of numerous clinical therapeutics, impacting their pharmacological and toxicological properties. The activation of protein kinase C (PKC) is shown to facilitate OAT1 internalization from cell surface to intracellular compartments and thereby reducing cell surface expression and transport activity of the transporter. The PKC-regulated OAT1 internalization occurs through ubiquitination, a process catalyzed by a E3 ubiquitin ligase, neural precursor cell expressed developmentally down-regulated 4–2 (Nedd4–2). Nedd4–2 directly interacts with OAT1 and affects ubiquitination, expression and stability of the transporter. However, whether Nedd4–2 is a direct substrate for PKC-induced phosphorylation is unknown.

**Results:**

In this study, we investigated the role of Nedd4–2 phosphorylation in the PKC regulation of OAT1. The results showed that PKC activation enhanced the phosphorylation of Nedd4–2 and increased the OAT1 ubiquitination, which was accompanied by a decreased OAT1 cell surface expression and transport function. And the effects of PKC could be reversed by PKC-specific inhibitor staurosporine. We further discovered that the quadruple mutant (T197A/S221A/S354A/S420A) of Nedd4–2 partially blocked the effects of PKC on Nedd4–2 phosphorylation and on OAT1 transport activity.

**Conclusions:**

Our investigation demonstrates that PKC regulates OAT1 likely through direct phosphorylation of Nedd4–2. And four phosphorylation sites (T197, S221, S354, and S420) of Nedd4–2 in combination play an important role in this regulatory process.

## Background

Organic anion transporters (OATs) are a group of transmembrane proteins specifically expressed at cellular barriers in multiple tissues, such as kidney, liver, intestine, placenta, brain, retina, and olfactory mucosa [1–4]. OATs play essential roles in mediating organic anion molecules across cell membranes, including a wide range of endogenous and exogenous molecules such as toxins and therapeutic drugs. Therefore, OATs are significant players in pharmacological responses including drug absorption, distribution, metabolism, and elimination [3, 5–9]. Several animal and clinical studies have been reported on the relationship between kidney diseases and renal OATs [3, 6]. While kidney diseases could directly affect the function and expression of renal OATs, damage to renal OATs could also alter various renal functions, leading to kidney disease progression [6]. For example, in a rat model of chronic kidney failure, OAT1 protein expression was reduced by 40% after oral administration of p-cresyl sulfate, a uremic toxin [10]. As an important member of the OAT family, OAT1 is expressed on the basolateral membrane of proximal tubule cells in kidneys. It facilitates the across-membrane transport of organic anion molecules from blood into proximal tubule cells, thus playing a significant role in the renal elimination of various therapeutic agents and metabolites [3, 5, 6, 9, 11, 12].

Protein phosphorylation is a post-translational modification where negatively charged phosphoryl groups are added to target proteins, which could affect function, expression, localization, or protein-protein interaction of these proteins [13–17]. Catalyzed by various protein kinases, phosphorylation stands as a significant regulatory mechanism for numerous membrane proteins including transporters, receptors, and channels [3, 13, 14, 18–22]. For instance, voltage-gated sodium channel was directly phosphorylated and modulated by both protein kinase A (PKA) and PKC [23]. Among the OAT family, OAT3 phosphorylation was significantly induced by PKA activation, and this increase was abrogated by PKA specific inhibitor [24, 25]. The enhanced phosphorylation of OAT3 was well correlated with increased protein expression and transport function, indicating PKA increased OAT3 expression and transport function through phosphorylating OAT3 directly [24–26]. Surprisingly, it has been demonstrated that PKC modulated OAT1 trafficking and transport activity without directly phosphorylating OAT1 itself, which led us to the search for potential target proteins of PKC-induced phosphorylation in this regulatory process [27].

Previous studies on PKC have revealed that activation of PKC promoted the attachment of a polyubiquitin chain to OATs, a process catalyzed by E3 ubiquitin ligase Nedd4–2 [14, 20, 28, 29]. Nedd4–2 protein consists of a HECT domain, a calcium/lipid binding domain, and four WW domains. The HECT domain at the C-terminus exerts the ubiquitin ligase activity of Nedd4–2, whereas the four WW domains often play essential roles in target recognition and binding. Our lab previously demonstrated that WW domains 3 and 4 within Nedd4–2 were responsible for interacting with OAT1 [30]. The increased ubiquitin level of OATs, catalyzed by Nedd4–2, led to the acceleration of OAT internalization from cell surface to intracellular compartments without changing its recycling rate, thereby decreasing cell surface expression and transport function of OATs [28, 29, 31–33]. Furthermore, long-term activation (> 4 h) of PKC resulted in increased degradation of total OATs in the cellular proteolytic system [30, 31, 34]. A recent study in rats revealed that PKCα expression was elevated after gentamicin-induced kidney injury, where both the membrane expression and function of renal Oat3 were decreased [35].

Despite the progress in understanding the regulatory mechanism of PKC on OATs, whether Nedd4–2 is a direct substrate for PKC-induced phosphorylation remains unknown. In the current study, our group investigated PKC effects on Nedd4–2 phosphorylation and identified potential phosphorylation sites on Nedd4–2, which would contribute to understanding the molecular mechanism underlying PKC-mediated regulation of OAT1.

## Results

### The effect of PKC on OAT1 transport function

**Fig. 1:**
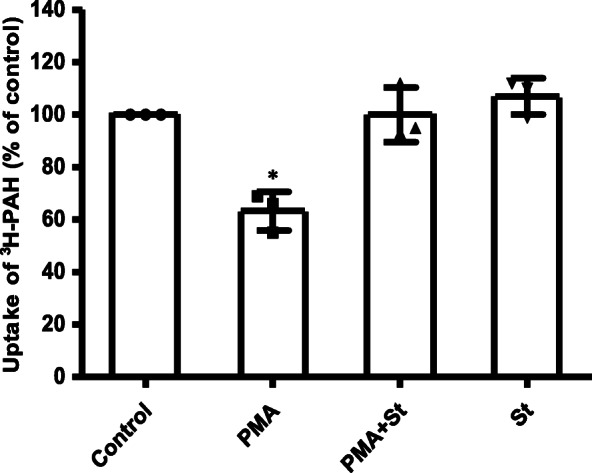
The effect of PKC on OAT1 transport function. OAT1-expressing COS-7 cells were treated for 1 h, with 10 μM PMA in the absence or presence of 2 μM staurosporine (St), or 2 μM St alone. 4-min uptake of [^3^H]-para-aminohippuric acid (PAH, 20 μM) was then measured at room temperature. Each data point represents OAT1-mediated transport only, after subtracting uptake values from cells transfected with empty vector. Uptake value in each group is expressed as a percentage of the uptake value measured in control cells. Values are mean ± S.D. (*n* = 3). *p < 0.05

We examined the effect of PKC activation on OAT1 transport function with cell-based uptake assay. OAT1-expressing COS-7 cells were treated for 1 h, with 10 μM phorbol 12-myristate 13-acetate (PMA) (PKC-specific activator) in the absence or presence of 2 μM staurosporine (St) (PKC-specific inhibitor). Then OAT1-mediated uptake of [^3^H]-para-aminohippuric acid (PAH) was measured at room temperature. The results (Fig. 1) showed that treatment with PMA reduced OAT1 transport activity by ~ 40% and staurosporine reversed the inhibitory effect of PMA, indicating PKC specificity of the regulation on OAT1. And at the same concentration, staurosporine itself did not affect OAT1 uptake function.

### The effect of PKC on OAT1 protein expression

**Fig. 2:**
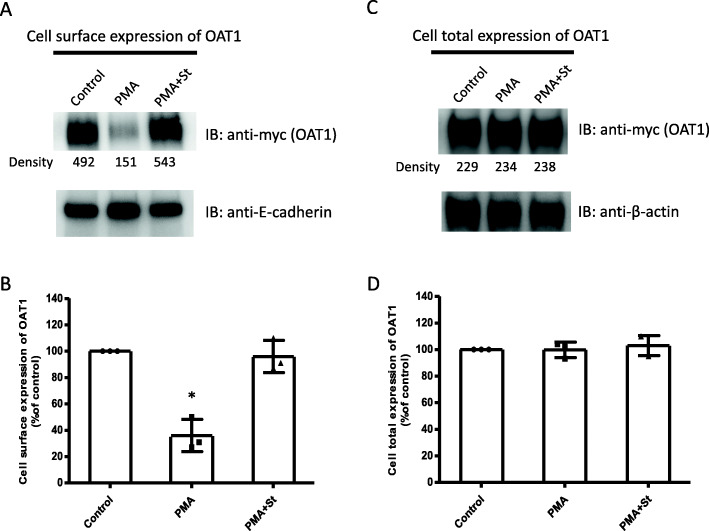
The effect of PKC on OAT1 expression. (A, top panel): cell surface expression of OAT1. OAT1-expressing COS-7 cells were treated for 1 h, with 10 μM PMA in the absence or presence of 2 μM staurosporine (St). Cells were then labeled with membrane-impermeable biotinylation reagent. Biotinylated cell surface proteins were then separated by streptavidin resin, followed by immunoblotting (IB) with anti-myc antibody (epitope myc was tagged to OAT1). (A, Bottom panel): The same immunoblot from the top panel was stripped and reprobed with anti-E-cadherin antibody to determine the expression of the cell surface protein marker E-cadherin. (B): Densitometry plot of results from (A) as well as from other repeat experiments. The values are mean ± S.D. (*n* = 3). *p < 0.05. (C, top panel): cell total expression of OAT1. (C, bottom panel): The same immunoblot from the top panel was stripped and reprobed with anti-β-actin antibody to determine the expression of β-actin, a protein marker for total cellular proteins. (D): Densitometry plot of results from (C) as well as from other repeat experiments. The values are mean ± S.D. (n = 3)

Subsequently, protein expression of OAT1 was assessed using a biotinylation method. OAT1-expressing COS-7 cells were treated for 1 h, with 10 μM PMA in the absence or presence of 2 μM staurosporine (St). Then cells were labeled with membrane-impermeable biotinylation reagent. After removing excessive biotinylation reagent, the cells were lysed and cell surface proteins were separated. Immunoblotting was utilized to detect myc-tagged OAT1 on cell surface or in total cell lysates. Our data (Fig. 2A, top panel & Fig. 2B) showed that PMA significantly reduced OAT1 expression on the cell surface while staurosporine reversed this decrease back to control level. In comparison, the expression of cell surface protein marker E-cadherin was not altered under the same treatment (Fig. 2A, bottom panel), suggesting that the changes of OAT1 surface expression were not due to the alterations of overall cell membrane proteins. Furthermore, the total cellular expression of OAT1 was not affected by the treatments (Fig. 2C & Fig. 2D).

### The effect of PKC on OAT1 ubiquitination

**Fig. 3:**
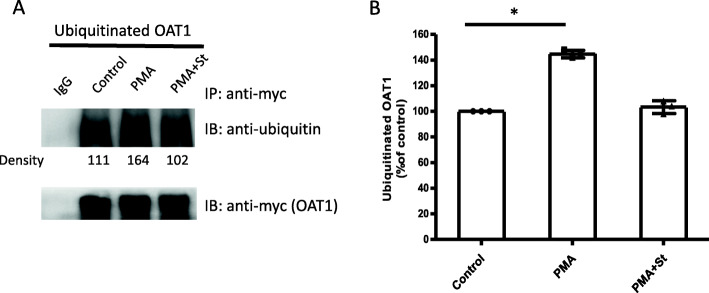
The effect of PKC on OAT1 ubiquitination**.** (A, top panel): OAT1 ubiquitination. OAT1-expressing COS-7 cells were transfected with wild type Nedd4–2. Transfected cells were incubated with 10 μM PMA for 1 h, with or without 2 μM staurosporine. Cells were lysed and OAT1 was immunoprecipitated (IP) with anti-myc antibody (epitope myc was tagged to OAT1). Normal mouse IgG was used as non-specific binding control (IgG). Then immunoblotting (IB) was performed with anti-ubiquitin primary antibody and anti-mouse secondary antibody. (A, bottom panel): The same immunoblot from the top panel was stripped and reprobed with anti-myc antibody to determine the total amount of OAT1 pulled down. (B): Densitometry plot of results from (A) as well as from other repeat experiments. The values are mean ± S.D. (*n* = 3). *p < 0.05

The ubiquitination level of OAT1 was examined after PKC activation. OAT1-expressing COS-7 cells were transfected with wild type Nedd4–2, and treated with 10 μM PMA for 1 h, with or without 2 μM staurosporine. Then immunoprecipitation with anti-myc antibody was conducted to purify myc-tagged OAT1 (mouse IgG as negative control), followed by immunoblotting with anti-ubiquitin antibody to detect ubiquitinated OAT1. Our results (Fig. 3A, top panel & 3B) showed that PMA significantly increased the level of OAT1 ubiquitination by ~ 40% and staurosporine reversed the increase to a similar level as that of the control group, while the amount of OAT1 pulled down remained equal among all groups (Fig. 3A, bottom panel), suggesting that the increased OAT1 ubiquitination was not due to the variation in the amount of OAT1 pulled down.

### The effect of PKC on Nedd4–2 phosphorylation

**Fig. 4:**
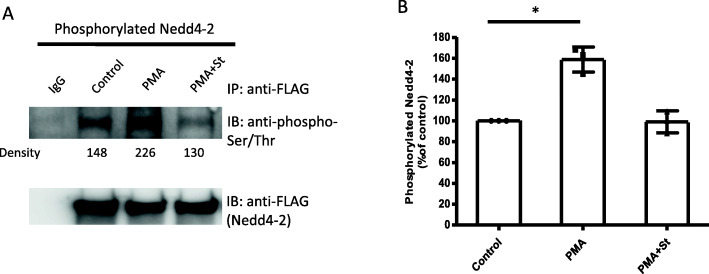
The effect of PKC on Nedd4–2 phosphorylation. (A, top panel): Phosphorylation of Nedd4–2. OAT1-expressing COS-7 cells were transfected with wild type Nedd4–2. Transfected cells were treated for 1 h, with 10 μM PMA in the absence or presence of 2 μM staurosporine (St). Cells were lysed and Nedd4–2 was immunoprecipitated (IP) with anti-FLAG antibody (epitope FLAG was tagged to Nedd4–2). Normal mouse IgG was used as non-specific binding control (IgG). Then immunoblotting (IB) was performed with anti-phospho-Ser/Thr primary antibody and anti-rabbit secondary antibody. (A, bottom panel): The same immunoblot from the top panel was stripped and reprobed with anti-FLAG antibody to determine the total amount of Nedd4–2 pulled-down. (B): Densitometry plot of results from (A) as well as from other repeat experiments. The values are mean ± S.D. (*n* = 3). *p < 0.05

Since it has been demonstrated that PKC regulated OAT1 function without direct phosphorylating the transporter itself [27], and Nedd4–2 played an important role in PKC-mediated OAT1 regulation [30, 32], the level of Nedd4–2 phosphorylation after PKC activation was investigated. OAT1-expressing COS-7 cells were transfected with wild type Nedd4–2 and treated with 10 μM PMA in the absence or presence of 2 μM staurosporine (St) for 1 h. After treatment, immunoprecipitation with anti-FLAG antibody was performed to purify FLAG-tagged Nedd4–2, followed by immunoblotting with anti-phospho-Ser/Thr antibody to detect phosphorylated Nedd4–2. Our results showed that PMA treatment significantly increased Nedd4–2 phosphorylation and staurosporine reversed the increase back to control level (Fig. 4A, top panel & Fig. 4B). And the amount of Nedd4–2 pulled down remained equal among all groups (Fig. 4A, bottom panel), suggesting that PKC-induced increase in Nedd4–2 phosphorylation was not due to the variation in the amount of Nedd4–2 pulled down.

### Identifying potential sites on Nedd4–2 for PKC-induced phosphorylation

To identify potential phosphorylation sites on Nedd4–2, OAT1-expressing COS-7 cells were transfected with wild type Nedd4–2 and treated with 10 μM PMA for 1 h. The cells were lysed and immunoprecipitated with anti-FLAG antibody to purify FLAG-tagged Nedd4–2. The purified Nedd4–2 was then separated by gel electrophoresis and stained with Coomassie blue. After comparing with the sample from cells transfected with control plasmid, a significantly up-regulated band at ~ 110 kDa was cut from the gel and sent for liquid chromatography-tandem mass spectrometry (LC-MS/MS) analysis. Four amino acid residues (Table. 1) with the most changes of phosphorylation in response to PKC activation were selected for the following site-directed mutagenesis on Nedd4–2.

**Table 1:** Potential PKC-induced phosphorylation sites on Nedd4–2

Phosphorylation Sites	Fold of Change
Thr197 (T197)	3.1
Ser221 (S221)	5.3
Ser354 (S354)	2.6
Ser420 (S420)	10

Table 1**.** Based on LC-MS/MS analysis of immunoprecipitated Nedd4–2, four amino acid residues with the most changes of phosphorylation in response to PKC activation were selected and listed above.

### The effect of PKC on phosphorylation of Nedd4–2 mutants

**Fig. 5:**
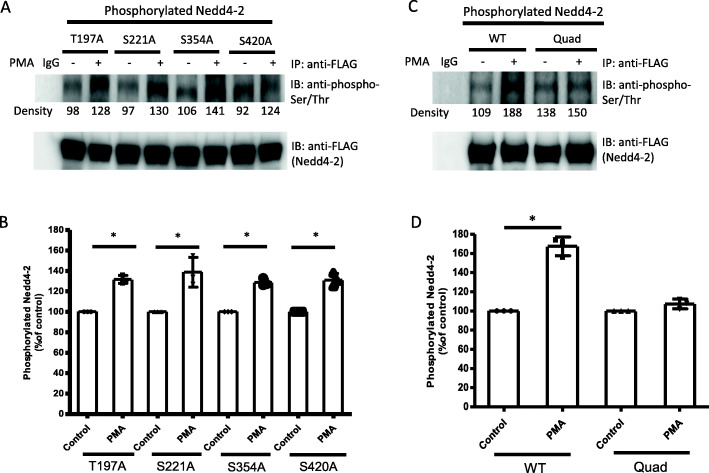
The effect of PKC on phosphorylation of Nedd4–2 mutants. (A, top panel): Phosphorylation of single mutants of Nedd4–2. OAT1-expressing COS-7 cells were transfected with individual single mutants of Nedd4–2: Thr197Ala (T197A), Ser221Ala (S221A), Ser354Ala (S354A), and Ser420Ala (S420A). Transfected cells were incubated with 10 μM PMA for 1 h. Cells were lysed and Nedd4–2 mutants were immunoprecipitated (IP) with anti-FLAG antibody (epitope FLAG was tagged to all Nedd4–2). Normal mouse IgG was used as non-specific binding control (IgG). Then immunoblotting (IB) was performed with anti-phospho-Ser/Thr primary antibody and anti-rabbit secondary antibody. (A, bottom panel): The same immunoblot from the top panel was stripped and reprobed with anti-FLAG antibody to determine the total amount of Nedd4–2 pulled-down. (B): Densitometry plot of results from (A) as well as from other repeat experiments. The values are mean ± S.D. (*n* = 3). *p < 0.05. (C, top panel): Phosphorylation of quadruple mutant of Nedd4–2. OAT1-expressing COS-7 cells were transfected with either wild type (WT) or quadruple mutant (Quad) of Nedd4–2. Transfected cells were incubated with 10 μM PMA for 1 h. Cells were lysed and Nedd4–2 (wild type and mutant) was immunoprecipitated (IP) with anti-FLAG antibody (epitope FLAG was tagged to all Nedd4–2). Normal mouse IgG was used as non-specific binding control (IgG). Then immunoblotting (IB) was performed with anti-phospho-Ser/Thr primary antibody and anti-rabbit secondary antibody. (C, bottom panel): The same immunoblot from the top panel was stripped and reprobed with anti-FLAG antibody to determine the total amount of Nedd4–2 pulled-down. (D): Densitometry plot of results from (A) as well as from other repeat experiments. The values are mean ± S.D. (n = 3). *p < 0.05

Based on the analysis of potential phosphorylation sites from mass spectrometry, we performed site-directed mutagenesis to generate four single mutants of Nedd4–2: Thr197Ala (T197A), Ser221Ala (S221A), Ser354Ala (S354A), and Ser420Ala (S420A). Their DNA sequences were all confirmed by Sanger sequencing. The phosphorylation of Nedd4–2 mutants under PKC activation was then examined. OAT1-expressing COS-7 cells were transfected with individual single-mutated Nedd4–2 and treated with 10 μM PMA for 1 h. The Nedd4–2 mutants were immunoprecipitated by anti-FLAG antibody, followed by immunoblotting with anti-phospho-Ser/Thr antibody to detect phosphorylated Nedd4–2 mutants. The results (Fig. 5A, top panel & Fig. 5B) showed that after PMA treatment, phosphorylation of all four single mutants were still increased by ~ 30%. Therefore, a quadruple mutant of Nedd4–2 (T197A/S221A/S354A/S420A) (Quad) was created by site-directed mutagenesis, where all four sites were simultaneously mutated. Phosphorylation of the quadruple mutant (Quad) after PMA treatment was not significantly increased comparing to the control group (Fig. 5C, top panel & Fig. 5D), suggesting the combination of four phosphorylation sites played a critical role in the PKC-induced phosphorylation of Nedd4–2.

### Quadruple mutant attenuated PKC effect on OAT1 transport function

**Fig. 6:**
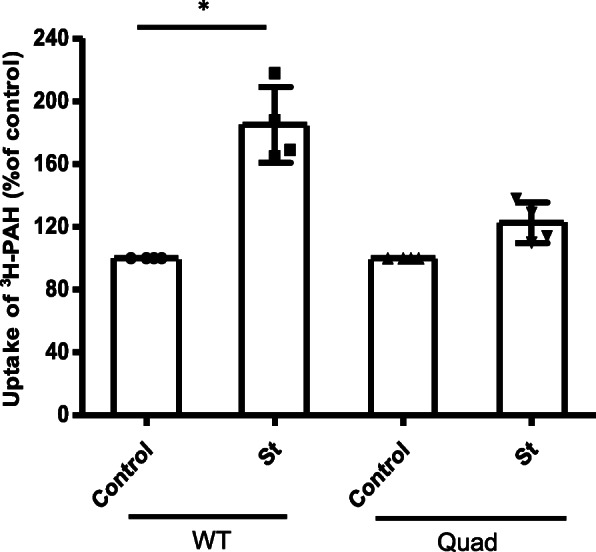
Quadruple mutant attenuated PKC effect on OAT1 transport function. OAT1-expressing COS-7 cells were transfected with either wild type (WT) or quadruple mutant (Quad) of Nedd4–2. Transfected cells were then treated with 5 μM staurosporine (St) for 1 h. 4-min uptake of [^3^H]-para-aminohippuric acid (PAH, 20 μM) was then measured at room temperature. Each data point represents OAT1-mediated transport only, after subtracting uptake values from cells transfected with empty vector. Uptake value in each group is expressed as a percentage of the uptake value measured in control cells. Values are mean ± S.D. (*n* = 4). *p < 0.05

Since the quadruple mutant of Nedd4–2 mostly blocked PKC-induced phosphorylation, the effect of PKC inhibition on OAT1 transport in cells transfected with quadruple mutant was further investigated. OAT1-expressing COS-7 cells were transfected with either wild type (WT) or quadruple mutated Nedd4–2 (Quad) and treated with PKC-specific inhibitor staurosporine (5 μM) for 1 h. After treatment, OAT1-mediated uptake of [^3^H]-PAH was measured at room temperature. Since activation of PKC has been shown to reduce OAT1 uptake function (Fig. 1), inhibition of endogenous PKC was expected to increase OAT1 uptake function. Our data (Fig. 6) showed that treatment of staurosporine (St) significantly increased OAT1 uptake by ~ 90% in cells expressing wild type Nedd4–2. And this stimulatory effect of staurosporine was largely prevented to only ~ 20% increase of uptake in cells expressing the quadruple-mutated Nedd4–2. These data suggest that PKC modulates OAT1 transport function partially through phosphorylating Nedd4–2 at these four sites.

## Discussion

OAT1 plays an important role in the renal clearance of numerous anionic drugs, which affects their therapeutic efficacy and toxicity. It also interacts with numerous endogenous metabolites and environmental toxins under physiological and pathological conditions [2, 3, 5, 6]. Thus, investigating the mechanisms of OAT1 regulation provides valuable information on new drug development and clinical drug use [7]. Previous studies from our lab revealed that unlike most of the protein kinases that regulate their protein substrates by directly phosphorylating them, PKC modulated OAT1 trafficking, expression, and transport function without directly phosphorylating the transporter itself [3, 14]. This surprising finding led us to search for substrates of PKC-induced phosphorylation in OAT1 regulation. In this study we identified ubiquitin ligase Nedd4–2 as the target of PKC-induced phosphorylation and phosphorylation of Nedd4–2 played an important role in the PKC-mediated regulation of OAT1, further elucidating the unique regulatory mechanism underlying OAT1 regulation by PKC.

We carried out this study in COS-7 cells, a cell line of kidney origin. Kidney is where native OATs are expressed [5]. This cell line has been widely used for investigating various renal transporters at molecular and cellular levels. First, this cell line lacks the endogenous expression of OATs, which allows us to express and study individual OATs without interference from other OAT family members. Second, the high transfection efficiency of COS-7 cells helps to improve the expression of OATs as well as other regulatory proteins after transfection, such as Nedd4–2 in this study. Furthermore, OATs expressed in the COS-7 system often exhibit similar properties and regulatory pathways compared to those in native tissues. Studies on OAT regulation performed in COS-7 cells would provide valuable information for future in vivo investigations on OAT function and regulation.

In the current study, we utilized small molecule activator and inhibitor to modulate PKC function and investigated their effects on OAT1. PMA, a phorbol ester, has been used extensively in biomedical research as a specific activator of PKC. It could also bind to some other proteins in less affinity, such as phospholipase D1 and chimaerins [20, 32, 36, 37]. Staurosporine is a classic protein kinase inhibitor with the highest potency for PKC with an IC_50_ value of ~ 3 nM, and is often used to block the activation of PKC from activators or upstream signaling pathways [30, 38–40]. Although it can also inhibit other kinases to a lesser extent, such as protein kinase G (IC_50_ of 18 nM) and myosin light-chain kinase (IC_50_ of 21 nM) [41]. In the current study, we cannot rule out the involvement of other kinases or proteins which might be inhibited by staurosporine to a certain degree.

The current study investigated the effects of PKC on OAT1 transport function, protein expression, ubiquitination, and Nedd4–2 phosphorylation. First, PKC activation was shown to significantly increase the amount of ubiquitinated OAT1 (Fig. 3A & B), a prerequisite for PKC-promoted acceleration of OAT1 internalization from cell surface to intracellular compartments [31, 34]. The ubiquitinated OAT1 from immunoblotting often appeared as smeared bands in a region between 100 and 150 kDa, with a relatively strong band centered at ~ 140 kDa. This is due to the fact that the polyubiquitin chains attached to a target protein often came in various sizes and structures [18, 28, 30, 34]. Thereby, anti-ubiquitin antibody could detect multiple bands of ubiquitinated OAT1. Second, our data showed that OAT1 uptake activity was specifically reduced by PKC activation (Fig. 1), which was accompanied by the decrease in OAT1 cell surface expression (Fig. 2A & B). And it was also observed that total cellular expression of OAT1 was not altered after PKC activation (Fig. 2C & D). These findings are consistent with the ubiquitination of OAT leading to an internalization of the transporter, resulting in a reduced surface expression and overall transport function. Based on our previous studies, a short-term activation of PKC, such as one hour, could lead to an internalization of ubiquitinated OAT to the intracellular compartments, where it can be de-ubiquitinated by deubiquitinating enzymes and then recycled back to cell surface. Under such a condition, OAT stability (the rate of degradation) is not affected, and therefore, its total amount does not change. That explains why the total OAT expression was not changed under our experimental conditions (Fig. 2C & D). Long-term PKC activation (> 4 h) results in prolonged OAT1 ubiquitination, and the excess amount of ubiquitinated OAT1 cannot be de-ubiquitinated quickly and therefore is targeted to proteasome for degradation, which eventually leads to the reduction of total amount of OAT1 [30]. Thus, in this study one-hour activation of PKC only reduced cell surface expression of OAT1 through increased ubiquitination and internalization, which contributed to the reduction of OAT1 transport function.

Since Nedd4–2 has been shown to directly interact with OAT1 and modulate its ubiquitination [30, 32], whether Nedd4–2 was a substrate of PKC-induced phosphorylation was subsequently investigated. The results from immunoprecipitation and immunoblotting revealed that PKC activation did increase Nedd4–2 phosphorylation significantly while PKC inhibitor staurosporine blocked the effect from PKC (Fig. 4A & B), indicating Nedd4–2 phosphorylation was PKC-specific. One step further, we employed LC-MS/MS analysis to identify the potential PKC-dependent phosphorylation sites on Nedd4–2. Four potential sites were identified based on the changes of their phosphorylation levels after PKC activation (Table 1). Additionally, when compared to the potential PKC phosphorylation sites predicted by NetPhos 3.1 software (Technical University of Denmark, Denmark), two of the sites (S354 and S420) selected from LC/MS analysis received high scores for potential PKC-dependent direct phosphorylation.

Then four single mutants were generated using site-directed mutagenesis to individually mutate these phosphorylation sites to alanine. Then the mutants were examined for their responses to PKC activation. Interestingly, the phosphorylation levels of the four single mutants were still induced by PKC activation (Fig. 5A & B). One of the possible mechanisms is that when one phosphorylation site is mutated, other phosphorylation sites could get phosphorylated to compensate for the loss of phosphorylation at that site, and therefore the overall phosphorylation of the single mutant protein would remain similar as that of wild type protein [15, 42]. Such possibility led us to create a quadruple mutant in which all four potential sites were simultaneously mutated to alanine. Indeed, this quadruple mutant significantly blocked the inducing effect of PKC on Nedd4–2 phosphorylation (Fig. 5C & D).

Furthermore, when we used staurosporine to suppress the endogenous PKC, OAT1 uptake function was increased by ~ 90% with cells expressing wild type Nedd4–2. This change was probably caused by the reduced level of phosphorylation on Nedd4–2 and its lower ubiquitin ligase activity. In contrast, OAT1 uptake was significantly weakened to an increase of ~ 20% with the quadruple mutant of Nedd4–2 expressed (Fig. 6), indicating PKC inhibition from staurosporine had little effect on the phosphorylation level and ligase activity of quadruple-mutated Nedd4–2. These data indicate that the four phosphorylation sites together play a critical role in PKC-dependent Nedd4–2 phosphorylation and downstream OAT1 regulation. Interestingly, without treatment, OAT1 uptake in cells expressing quadruple-mutated Nedd4–2 was higher than that that in cells with wild type Nedd4–2, the quadruple mutant itself increased OAT1 transport function to some extent. There are possibly some independent effects of staurosporine and the quadruple-mutated Nedd4–2 on OAT1 transport function which requires further investigation. In addition to the phosphorylation of Nedd4–2, we also observed that PMA (PKC activator) significantly increased the auto-ubiquitination of Nedd4–2 (data not shown), indicating that the auto-ubiquitination of Nedd4–2 might be involved in regulating its ubiquitin ligase activity and interaction with OAT1. Such situation has recently been reported on Nedd4–1, another ubiquitin ligase in the Nedd family, in which the auto-ubiquitination of Nedd4–1 was required for its ubiquitin ligase activity and interactions with both ubiquitin-specific protease 13 and class III phosphoinositide 3-kinase vacuolar protein sorting 34 [43].

For the next step, we are planning to further investigate the effects of PKC on Nedd4–2 phosphorylation, auto-ubiquitination, and interaction with OAT1, as well as the altered expression and function of OAT1 in animal models. These studies would greatly advance our understanding of the in vivo regulation of OAT1 by Nedd4–2 and PKC.

## Conclusions

The current study demonstrates that PKC regulates OAT1 likely through direct phosphorylation of Nedd4–2. And four phosphorylation sites (T197, S221, S354, and S420) on Nedd4–2 in combination play an important role in this regulatory process.

## Methods

### Materials

Parental COS-7 cells were purchased from ATCC (Manassas, VA). [^3^H]-labeled *para*-aminohippuric acid was purchased from PerkinElmer (Waltham, MA). Membrane-impermeable biotinylation reagent sulfo-NHS-SS-biotin, streptavidin agarose beads, protein G-agarose beads, and protease inhibitor cocktail were obtained from Pierce Biotechnology (Rockford, IL). Plasmid for wild type human Nedd4–2 was generously provided by Dr. Peter Snyder from University of Iowa (Iowa City, IA). Site-directed mutagenesis kit was purchased from Agilent Technologies (Santa Clara, CA). Mouse anti-myc and anti-β-actin antibodies were purchased from Roche (Indianapolis, IN). Mouse anti-E-cadherin antibody was purchased from Abcam (Cambridge, MA). Mouse anti-FLAG antibody and anti-FLAG M2 affinity beads were purchased from Sigma-Aldrich (St. Louis, MO). Rabbit anti-phospho-Ser/Thr antibody was obtained from Cell Signaling Technology (Danvers, MA). Phorbol 12-myristate 13-acetate (PMA), staurosporine (St), and all other reagents were purchased from Sigma-Aldrich (St. Louis, MO).

### Cell culture and transfection

hOAT1-expressing COS-7 cells were maintained in Dulbecco’s modified Eagle’s medium (DMEM) containing 10% fetal bovine serum and 0.4 mg/mL of G418 (Invitrogen, Carlsbad, CA) at 37 °C in 5% CO_2_. hOAT1 was tagged with a myc-tag to its carboxyl terminus for immunoprecipitation and immunodetection of the transporter. Wild type and mutant Nedd4–2 were tagged with FLAG-tag for immunoprecipitation and immunodetection. Lipofectamine 2000 (Invitrogen, Carlsbad, CA) was used for transient transfection of protein-expression plasmids following the manufacturer’s instructions. Forty-eight hours after transfection, the cells were used for following experiments.

### Liquid chromatography-tandem mass spectrometry (LC-MS/MS)

Each gel band was subjected to reduction with 10 mM DTT for 30 min at 60 °C, alkylation with 20 mM iodoacetamide for 45 min at room temperature in the dark, and digestion with trypsin (Thermo Scientific, San Jose, CA), and incubated overnight at 37 °C. Afterwards, peptides were extracted twice with 5% formic acid plus 60% acetonitrile and dried under vacuum. Samples were analyzed using a Q Exactive HF tandem mass spectrometer coupled to a Dionex Ultimate 3000 RLSCnano System (Thermo Scientific, San Jose, CA). Samples were loaded on a fused silica trap column (Acclaim PepMap 100, 75μmx2cm, ThermoFisher Scientific). After washing for 5 min at 5 μl/min with 0.1% TFA, the trap column was brought in-line with an analytical column (Nanoease MZ peptide BEH C18, 130A, 1.7 um, 75μmx250mm, Waters) for LC-MS/MS. Peptides were eluted using a segmented linear gradient from 4 to 90% buffer B (0.08% formic acid, 80% ACN). Mass spectrometry data was acquired using a data-dependent acquisition procedure with a cyclic series of a full scan with resolution of 120,000 followed by MS/MS (HCD, relative collision energy 27%) of the 20 most intense ions and a dynamic exclusion duration of 20 s. The peak list from LC-MS/MS was generated by Thermo Proteome Discoverer v. 2.1 and searched against Uniprot human proteome reference plus a database of common lab contaminants. Search parameters were as follows: fragment mass error: 20 ppm, parent mass error: +/− 7 ppm; fixed modification: carbamidomethylation on cysteine; flexible modifications: oxidation on methionine; phosphorylation on serine, threonine and tyrosine. Interested phosphorylation sites were manually inspected and identified.

### Site-directed mutagenesis

The plasmids of Nedd4–2 mutants (single and quadruple) were generated in our laboratory using a QuickChange site-directed mutagenesis kit from Agilent Technologies (Santa Clara, CA), followed by the confirmation of DNA sequences by Sanger sequencing.

### Measurement of OAT1-mediated transport function

The uptake solution consisted of phosphate-buffered saline with 1 mM CaCl_2_, 1 mM MgCl_2_, and 20 μM [^3^H]-*para*-aminohippuric acid. 1E+ 5 COS-7 cells were plated in each well of 48-well plates and grown overnight (20–24 h) for uptake assay. The uptake solution was added to cells for a 4-min uptake at room temperature, then the uptake was terminated by removing the uptake solution and washing the cells twice with ice-cold phosphate-buffered saline. The cells were then lysed in 0.2 N NaOH for 30 min, neutralized with 0.2 N HCl, and subjected to liquid scintillation counting by a liquid scintillation counter (Beckman LS6500). Uptake activity was expressed as a percentage of the uptake measured in control cells. Uptake values were adjusted for passive diffusion measured in mock cells (parental COS-7 cells transfected with empty plasmids). Uptake data from each group were also normalized based on the average total protein concentrations in each group.

### Cell surface Biotinylation

The amount of OAT1 on the plasma membrane was assessed using the membrane-impermeable biotinylation reagent, sulfo-NHS-SS-biotin. Briefly, live cells were incubated with NHS-SS-biotin for 20 min twice on ice. Afterward, the cells were washed with phosphate-buffered saline with 1 mM CaCl_2_, 1 mM MgCl_2_, and 100 mM glycine twice to quench the unreacted sulfo-NHS-SS-biotin. The cells were then lysed in lysis buffer (10 mM Tris-HCl, 150 mM NaCl, 1 mM EDTA, 0.1% SDS, 1% Triton X-100, and 1% protease inhibitor cocktail). After lysis, the cell lysates were centrifuged at 16,000×g 4 °C for 20 min, followed by the addition of streptavidin-agarose beads to the supernatant to isolate cell surface proteins. After overnight incubating with streptavidin beads at 4 °C, cell surface proteins were released from beads and separated with gel electrophoresis, followed by immunoblotting using anti-myc antibody (1:5000 diluted) to detect myc-tagged OAT1 on cell surface. E-cadherin (1:5000 diluted) was also examined as a cell surface protein marker. β-actin (1:5000 diluted) was measured as a cell total protein marker.

### Immunoprecipitation

Cells were lysed with lysis buffer (10 mM Tris-HCl, 150 mM NaCl, 1 mM EDTA, 0.1% SDS, 1% Triton X-100, 1% protease inhibitor cocktail, and 1% phosphatase inhibitor cocktail). After centrifugation at 16,000×g 4 °C for 20 min, the cell lysates were precleared at 4 °C with protein G agarose beads for 4 h to reduce nonspecific binding. The precleared lysates were then mixed with antibody attached protein G agarose beads and incubated overnight at 4 °C. On the following day, the beads were washed with lysis buffer for three times. Proteins bound to the beads were released and denatured by 2x laemmli buffer (ThermoFisher Scientific, Waltham, MA), followed by gel electrophoresis and immunoblotting.

### Gel electrophoresis and immunoblotting

Denatured protein samples were separated on 7.5% polyacrylamide gels and electroblotted onto polyvinylidene difluoride (PVDF) membranes. Then PVDF membranes were washed in PBS, blocked with 5% nonfat milk in PBS plus 0.1% Tween-20, and incubated with primary antibodies (anti-myc 1:5000; anti-ub 1:500; anti-FLAG 1:5000; anti-Phospho-S/T 1:1000) diluted in 3% nonfat milk in PBS plus 0.1% Tween-20 at 4 °C overnight, and subsequently incubated with horseradish peroxidase–conjugated secondary antibodies (anti-mouse HRP 1:5000; anti-rabbit HRP 1:2000). SuperSignal West Dura Extended Duration Substrate kit (Pierce Biotechnology, Rockford, IL) and ChemiDoc Imaging System (Bio-Rad, Hercules, CA) were utilized to detect the chemiluminescent signals from target proteins. Western blot images were selected and processed using Image Lab software (Bio-Rad, Hercules, CA). In brief, non-saturated and specific protein bands were selected based on target protein size and negative control. After proper adjustment of brightness and intensity on full images, regions of interest were cropped, leaving enough space around target bands. The scanning densitometry was performed to quantify the non-saturated, immunoreactive protein bands, where same-sized areas were selected and analyzed by the Volume Tool within Image Lab software. Background signal was subtracted from the values of quantified bands, and relative protein expressions were presented as percentage of those in control groups.

### Data analysis

Each experiment was repeated independently for a minimum of three times, and multiple repeats were used for statistical analysis. Between two groups, statistical analysis was performed using Student’s paired t-test. Among multiple groups, one-way ANOVA and Post Hoc Tukey’s test were applied using GraphPad Prism software (GraphPad Software Inc., San Diego, CA). A *p*-value < 0.05 was considered statistically significant.

## Data Availability

All data generated or analyzed during this study are included in this published article.

## Abbreviations

IB: immunoblotting
IP: immunoprecipitation
LC-MS/MS: liquid chromatography-tandem mass spectrometry
Nedd4–2: neural precursor cell expressed developmentally downregulated 4–2
OAT1: organic anion transporter 1
OATs: organic anion transporters
PAH: para-aminohippuric acid
PMA: phorbol 12-myristate 13-acetate
PKA: protein kinase A
PKC: protein kinase C
St: staurosporine
